# The dynamics of physics in PET

**DOI:** 10.1186/2197-7364-1-6

**Published:** 2014-06-03

**Authors:** Charles C Watson

**Affiliations:** Siemens Healthcare, 810 Innovation Drive, Knoxville, TN 37932 USA

**Keywords:** Physics, PET, Nuclear medicine, Instrumentation

## Abstract

From a technical perspective, there are fundamentally two forces driving the evolution of instrumentation in positron emission tomography (PET) and nuclear medicine generally: clinical needs and technical innovation. This essay considers some of the dynamics of these forces as they act on physics-related developments in PET and suggests that progress will be greatest if these differing motivations are kept in balance as the field evolves.

## Background

In the context of positron emission tomography (PET), and nuclear medicine generally, the term ‘physics’ has come to refer to far more than just the underlying physical principles of the generation, transport, and detection of nuclear radiation; it encompasses many aspects of the delivery and uptake of radioactive tracers, instrumentation design, signal processing, data corrections, image reconstruction, and quantitative analysis. The scope of the historical and future development of this field, as eloquently laid out in the four inaugural papers in this journal [1–4], is truly remarkable and challenging to comprehend fully. In trying to understand the dynamics that govern this development, I find it helpful to analyze them in terms of two motivating forces that these authors have described: the needs of clinical applications and the possibilities of technical innovations. These are, of course, just the endpoints of the spectrum of scientific motivations for change in nuclear medical physics, and most advances will be influenced to a greater or lesser extent by both.

## Main text

Clinically driven development is that which is motivated primarily by the desire to satisfy a specific clinical need for which no adequate solution currently exists. The introduction of multi-bed, whole-body imaging, for example, addressed the issue of diagnosing the metastatic spread of cancer [5]. The initial implementations of attenuation, scatter, and prompt-gamma corrections in PET responded to the need for quantitation of tracer uptake. The development of specialized systems for breast and prostate cancer imaging and high-resolution head scanners for neurological studies are all examples of such clinically motivated progress. Recently, the advent of tracers enabling beta-amyloid plaque imaging in the brain has sparked the implementation of new attenuation correction and image analysis techniques designed specifically to support screening for Alzheimer’s Disease [6].

Physics-driven development, on the other hand, is that which is motivated more by the desire to improve the technology or performance of an imaging (or other) system in general rather than to achieve a specific clinical goal, although it is nevertheless ultimately justified by the value it brings to established clinical applications or novel ones that it enables. The transition from 2D (with inter-plane septa) to 3D (without septa) PET imaging in the 1990s, for example, involved a long process of trying to understand the benefits of 3D and optimize its performance in general-purpose whole-body scanners [7]. This optimization was frequently based on a physical rather than a clinically derived metric [8]. Similarly, the introductions of LSO/LYSO scintillators, iterative reconstruction algorithms, and time-of-flight (TOF) measurement were all motivated by the desire to improve imaging performance generally. Today, we are witnessing the first wave of a similarly important technological advance: the replacement of conventional photomultiplier tubes with solid-state photomultipliers, which holds great promise to reduce the size and increase the performance of PET scanners [9, 10].

A distinctive characteristic of physics-driven innovation is that it tends to be more risky than clinically driven development; its value may not be as obvious from the outset. As hard as it may be to believe today, the development and introduction of PET/CT was slowed by the considerable initial skepticism with which the concept was met. Equipment manufacturers were unsure at first of its commercial potential and hesitant to invest heavily in it. Many in nuclear medicine doubted its clinical utility and cost effectiveness. It was not until the value of the fused PET and CT images and superiority of the CT-based attenuation correction became obvious from actual clinical use that the landslide of its adoption began. The PET/MR is arguably an even more technically and clinically complex system than PET/CT, and thus, one whose ultimate clinical value is even more difficult to foresee. Sometimes, technologies simply fail to meet expectations, such as the ‘wobble’ feature on many early PET gantries designed to improve spatial resolution, but whose benefits were eventually judged not worth the inherent problems of increased complexity and noise, and reduced mechanical reliability. A hybrid PET/SPECT system [11] developed in the late 1990s, with innovative LSO/NaI phoswich detectors and novel coincidence point source transmission capability, never took off clinically due partly to the fact that compelling applications for it never emerged.

On the other hand, it’s important to recognize that even when a new physics-driven development fails to be widely adopted clinically, it may still lead to important advances. The first prototype of the PET/CT that provided proof-of-principle data was inspired by, and constructed from, technology and components developed for an innovative partial-ring rotating tomograph that itself never made the commercial mainstream [12]. Likewise, the first incarnation of time-of-flight (TOF) PET based on CsF and BaF_2_ detectors ultimately failed in its competition with more sensitive BGO-based systems. Yet, the extensive research and development of TOF for these early systems laid a solid foundation for its rapid adoption once enabling LSO/LYSO detector technology matured and became widely available [13].

Another important aspect of the dynamics of PET physics is the synergism between technologies developed more or less independently that can lead to unanticipated advances. An interesting example is that of attenuation correction (AC). Prior to PET/CT, AC was based almost exclusively on transmission measurements made with radioactive sources. With the advent of CT-based AC, such techniques became obsolete. Recently, however, there has been renewed interest in transmission measurements due to several complimentary developments: New joint emission-attenuation reconstruction algorithms permit extraction of a great deal of attenuation information from emission-only [14, 15] or simultaneous emission-transmission [16] data. Positron beams offer a mechanically simple means for injecting transmission sources into the field of view of integrated PET/MR systems [16]. TOF measurements allow useful discrimination of emission and transmission data in simultaneous scans [17]. Furthermore, it has been increasingly realized that the needed AC information may be derived from multiple sources used together: emission data, partial transmission data, and segmented MR images, for example [15, 16]. Such combinations of technologies open up new possibilities not envisioned a decade ago. This type of synergistic advance seems more likely to occur in the context of physics-driven developments than in clinically driven ones.

## Conclusions

From my perspective in commercial PET development, it seems that the history of PET cameras over the past 20 years has largely been driven by efforts to make better images of [^18^F]-fluoro-deoxy-glucose (FDG). Because the clinical target was so slowly moving, it provided a great opportunity for optimizing the physics of this part of PET. But perhaps, it could be said that as a consequence, we in the medical imaging community did not spend our resources as well as we might have to bring progress to PET or nuclear medicine as a whole. The picture of the future painted in the inaugural papers in this journal [1, 4] is quite different: FDG’s role will diminish, and we will see the evolution of nuclear medicine become increasingly driven by the clinical need to provide ever more sensitive and precise techniques to characterize and influence the molecular biology of disease in a variety of new and different ways, using an increasing array of biomarkers, multi-parametric methods, and radionuclide therapies. This will undoubtedly be a healthy development for the field; but at the same time, I think it is important to keep in mind that PET and other nuclear medical technologies are still very far from achieving their full technical potential. Tremendous fundamental advances in time, energy, and spatial resolutions; sensitivity; data corrections; reconstruction; and information extraction still remain to be realized [1, 4]. As we make the transition to a future of more varied and demanding clinical applications, it will be important to maintain a proper balance between the contributions from clinically oriented physics research (in the broadest sense of this term) on one side of the spectrum, and the technology-oriented physics research on the other. Clearly, we need to focus on the more promising new clinical applications to guide us in the most efficient use of our resources, but it would be unfortunate if in doing this we under-invested in the type of non-application-specific, physics-driven, technological innovations, which, though sometimes risky, have often proven highly rewarding.

One requisite for achieving such balance is efficient communication about new requirements and potential solutions among those working across the spectrum. This will be challenging, but I am hopeful that this new physics-focused, open-access journal will play a very useful role in advancing that goal.
